# Head and neck INI1-deficient carcinoma without primary: a case report

**DOI:** 10.1186/s13256-023-04214-3

**Published:** 2023-11-17

**Authors:** Antoine Dubray-Vautrin, Wahib Ghanem, Laurence Bozec, Julie Gonin, Olivier Choussy

**Affiliations:** 1 Department of Oto-Rhino-Laryngology, Head & Neck Surgery, Institut Curie, 26 Rue D’Ulm, 75005 Paris, France; 2 Department of Oncology, Institut Curie, Saint Cloud, France; 3 Departement of Pathology, Institut Curie, Saint-Cloud, France

**Keywords:** SMARCB1 protein, Case report, Carcinoma, Unknown primary

## Abstract

**Background:**

SMARCB1, also known as INI1, is a member of a large protein complex involved in chromatin remodeling and thus the regulation of gene expression. It is located on chromosome 22q11.2. SMARCB1 tumors have been found in various locations, including the sinonasal region, gastrointestinal tract, central nervous system (in atypical teratoid and rhabdoid tumors), and perirenal region (in malignant rhabdoid tumors) in both adults and children.

**Case presentation:**

We describe here the first case in the literature of an INI1-deficient neck carcinoma without a primary tumor managed with surgical therapy and neck dissection in a young Caucasian woman of 29 years old, followed by chemotherapy before radiotherapy, with regional control after 18 months of follow-up. Histologic analysis showed an undifferentiated carcinoma without glandular or epidermoid differentiation. Biomolecular analysis of the tumor revealed a homozygous deletion of the SMARCB1 gene on RNA sequencing.

**Conclusion:**

Research of INI1 deletion should be performed for undifferentiated carcinoma of young patients because of possibilities of molecular therapies such as autophagy inhibitors or proteasome inhibitors could be used in clinical trials.

## Background

SMARCB1 is a tumor suppressor gene located on 22q11.2, which codes for a core subunit protein of the ATP-dependent SWI/SNF chromatin remodeling complex. Loss-of-function mutations in SMARCB1 have been identified as a cause of various histologic tumors [1], including epithelioid sarcomas, schwannomatosis, synovial sarcomas, and myoepithelial carcinomas. These tumors have been reported in different locations, such as the sinonasal region [1], vulvar region, gastrointestinal tract [2], atypical teratoid/rhabdoid tumors of the central nervous system, and perirenal malignant rhabdoid tumors in both adults and children [3, 4]. SMARCB1-deficient tumors occur in children and adults with a range of 0 to 80 years old. The histologic polymorphism of this tumor is significant, ranging from benign to highly aggressive with poor prognosis, even with aggressive multimodality therapy [5].

We hereby described the unusual case of a young woman with a neck node metastasis of an INI1-deficient carcinoma. No primary cancer was found after clinical, radiological, histological, and biomolecular examinations.

## Case presentation

**Fig. 1:**
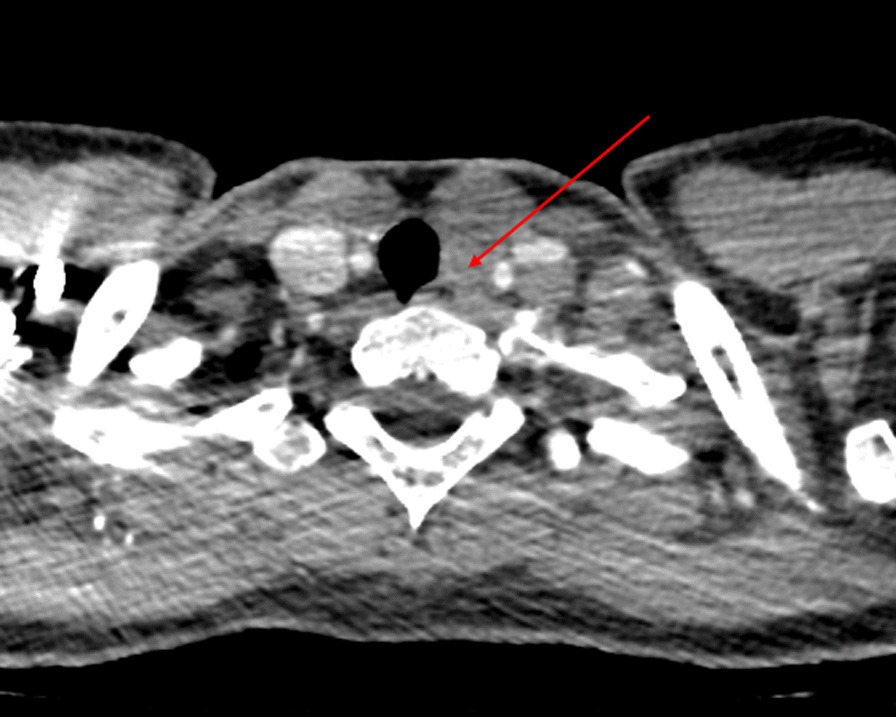
Neck CT scan showing a laterotracheal node of 4 cm proximate to the esophagus

A 29-year-old Caucasian woman presented at a tertiary hospital center with a 6-month history of dysphonia. She had no significant personal, familial, or genetic medical history, no alcohol or tobacco use, and no reported toxic consumption. Physical examination revealed a 2 cm left group VI adenomegaly upon neck palpation. Endoscopic examination revealed left vocal cord palsy. Neck computed tomography (CT) scan confirmed a 4 cm group VI lymph node (Fig. 1). Fine needle aspiration indicated a malignant lesion, initially suspected to be melanoma. Dermatologic, sinonasal, thyroid, and breast examinations did not reveal any tumors, and both breast echo tomography and MRI examinations were negative.

**Fig. 2:**
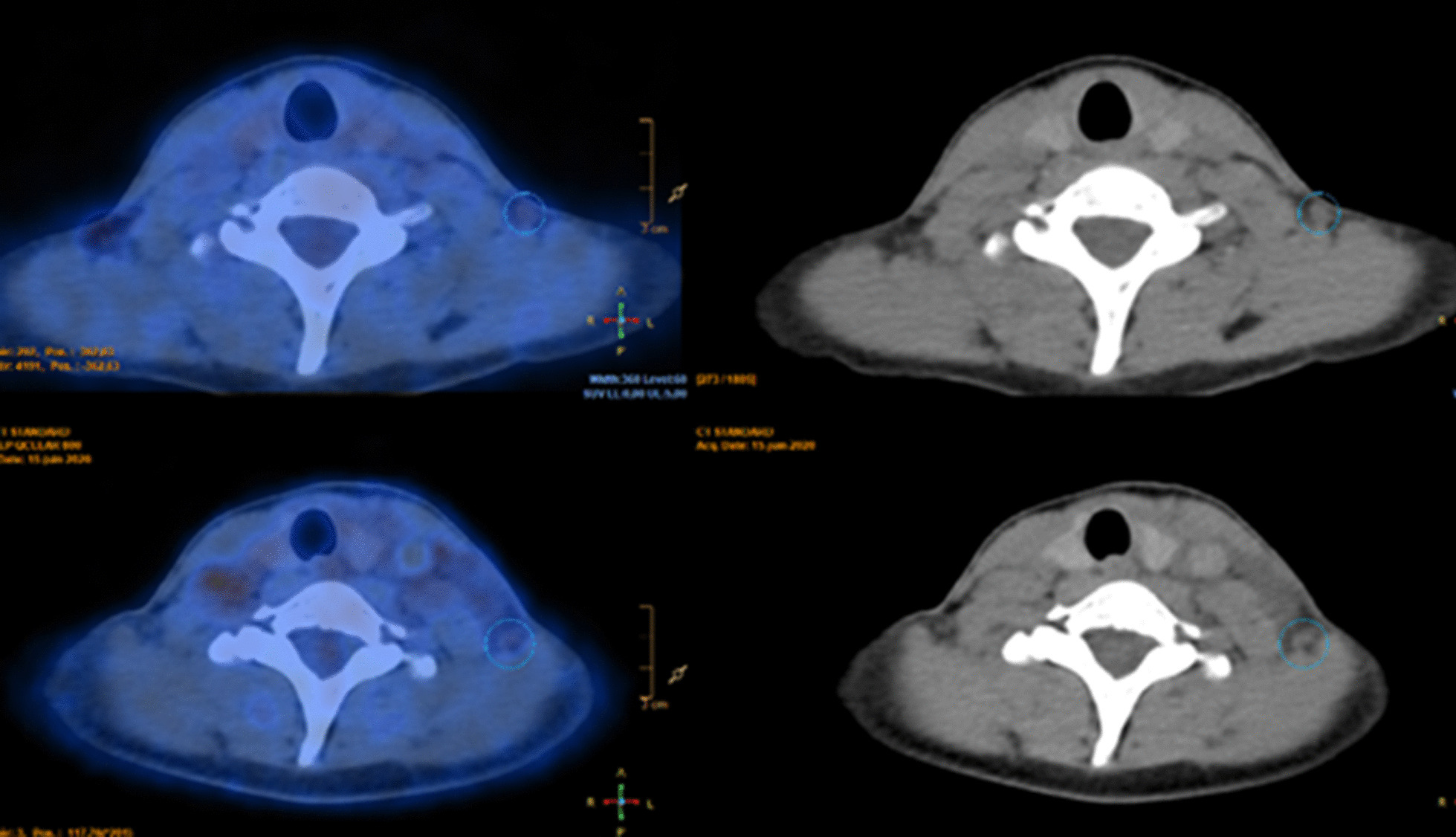
FDG PET–CT (computed tomography) with no primary and a slight hypermetabolism of left IV group after initial surgery

A group VI and group IV neck dissection was initially performed, sacrificing the left inferior laryngeal nerve. Complementary 18F-fluoro-2-deoxyglucose (FDG) positron emission tomography (PET)/computed tomography was performed, but no primary tumor was identified. Only residual lymph node hypermetabolism in the left group IV was described (Fig. 2).

**Fig. 3:**
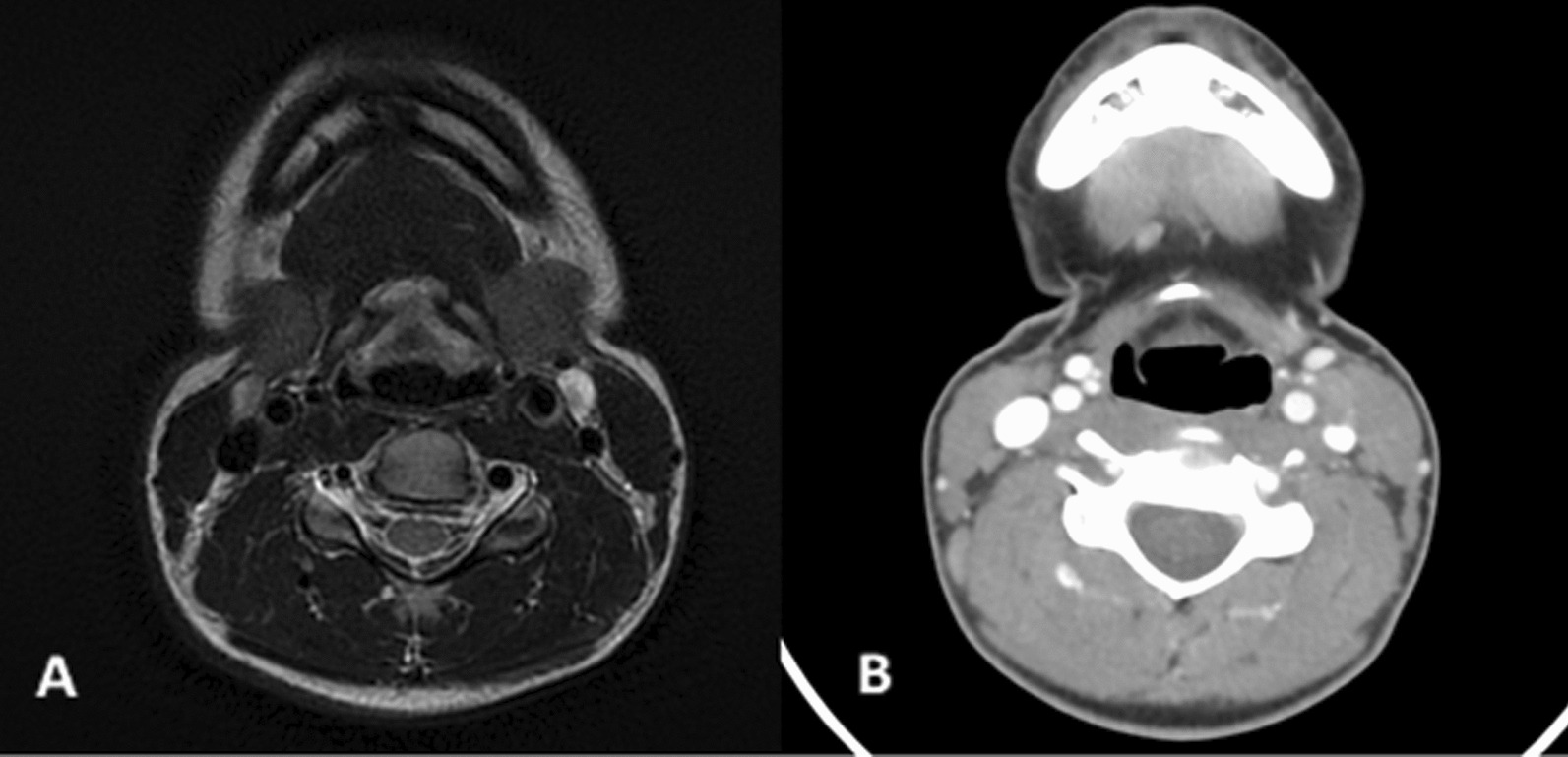
**A** Neck MRI (T2 fat saturation) and **B** injected neck CT scan confirmed the presence of supracentimetric bilateral neck node disease

The patient underwent follow-up imaging with a head, neck, and abdominal CT scan and an MRI 6 weeks after the initial surgery. The imaging revealed the presence of 15 mm and 20 mm neck nodes in the left IIa and III groups, as well as a 15 mm adenomegaly in the right group III neck area. No primary tumor was detected in the nose, sinus, oropharynx, salivary gland, or kidney in this radiological description (Fig. 3).

## Treatment, outcome, and follow-up

In a tumor board decision, we collectively decided to perform a complementary bilateral neck dissection of group I, IIa, IIb, III and IV. After definitive histological analysis, the national committee of adenocarcinoma without a primitive (ACUP) confirmed the need for adjuvant therapy due to left node recurrence, 4 weeks after the second surgery. The patient was treated with two cycles of chemotherapy, including CARBOPLATINE and paclitaxel at a dose of 175 mg/m^2^. After two cycles, neck CT scan confirmed the absence of neck progression. We completed chemotherapy with six cycles and performed adjuvant radiotherapy–chemotherapy, administering a total dose of 66 Gy in 33 fractions, with cisplatin (100 mg/m^2^) given on day 1, 22, and 43. After performing molecular analysis, we confirmed the biallelic inactivation of SMARCB1. We conducted a complementary MRI of the kidney to rule out a malignant tumor of the kidney as the primary source. Clinical and radiological evaluations at 3-, 6-, and 18-months post-treatment confirmed local and distant disease control. Histologic findings after neck dissection confirmed an INI1-deficient neck tumor without a primary tumor source.

## Pathological findings

### Macroscopic analysis

On first lymphadenectomy involving central and group IV neck dissection, 23/28 nodes were metastatic with extranodal extension. On the second lymphadenectomy, 2/21 left side nodes were metastatic, one with extra-nodal extension. Right lymph nodes were all negative (*n* = 43). Submandibular glands were free of disease.

### Microscopic analysis

**Fig. 4:**
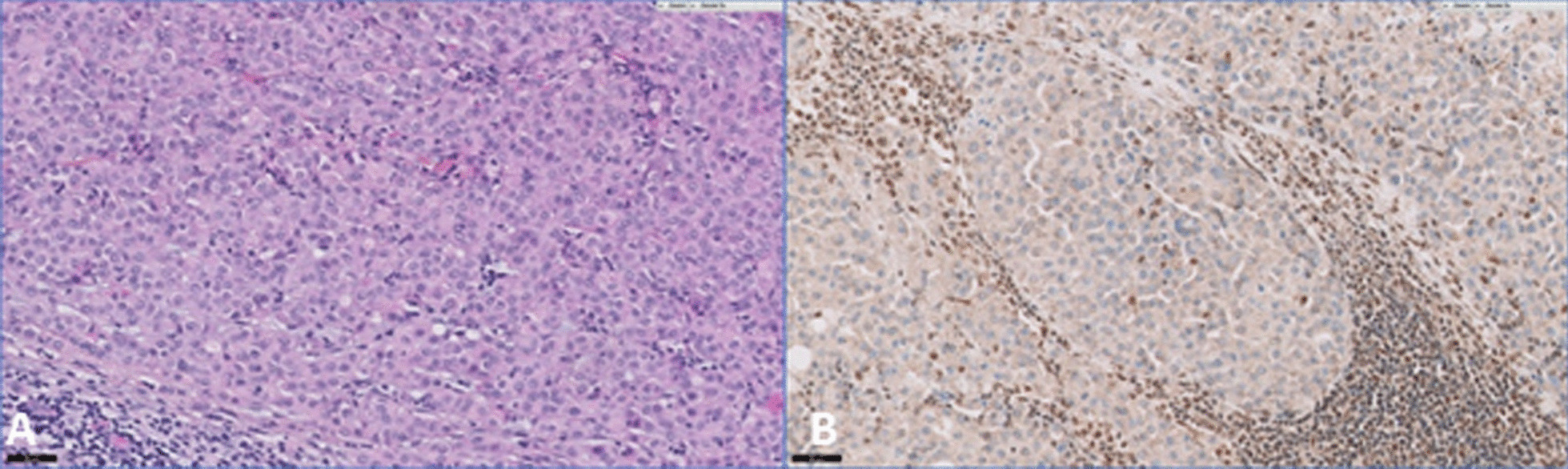
**A** Histological sections show sheets of epithelioid cells with eosinophilic cytoplasm and round nuclei with nucleolus (HES ×200 magnification). **B** Loss of INI1 (BAF47) nuclear staining in tumor cells with preserved expression in normal residual lymphocytes (×200 magnification)

Morphology on hematoxylin–eosin–saffron (HES) stains showed sheets of epithelioid cells with abundant eosinophilic cytoplasm and round nuclei with prominent nucleolus. No glandular or epidermoid differentiations were detected, and no pigment was detected. Background contained mucoid deposit. Necrotic areas were noted (Fig. 4).

### Histochemistry and bio molecular analysis

Immunostains showed expression of pancytokeratin AE1/AE3, cytokeratin 19 without concomitant expression of CK7, CK20, or CK5/6. Epithelial Membran Antigen (EMA) and epithelial cadherin were positive. Intense chromogranine A expression was observed without expression of other neuroendocrine markers (synaptophysin and CD56 or calcitonin). Melanocytic markers (S100, SOX 10 HMB45, and melan A) were negative, as well as estrogen, progesterone, and androgen receptors, p63, p40, PAX8, TTF1, SAll4, GCDFP15, EGFR, and thyroglobulin. P16 was positive in 30% cells with a patchy staining. HPV testing was negative. Interestingly, HER2 showed a positive cytoplasmic staining. FISH HER2 was negative. INI1 (BAF47) testing showed nuclear loss in tumor cells, with preserved nuclear staining in lymphocytes (Fig. 4). Molecular findings confirmed the biallelic inactivation of the SMARCB1 gene with a homozygote deletion on next-generation sequencing (NGS) and a fusion transcript SMARCB1/ RP6-65G23.3 on RNA sequencing (RNAseq).

## Discussion

SMARCB1 (INI1) is a member of a large protein complex involved in chromatin remodeling, thus regulating gene expression. It is located on chromosome 22q11.2 [6]. Loss of SMARCB1 expression resulting from deletions/mutations has emerged as a defining diagnostic feature in various neoplasms in both children and adults. It is found in malignant atypical teratoid (AT)/rhabdoid tumors (RT) of childhood and can also be observed as an additional alteration on a preexisting background of genetic alterations [2, 7–9]. Other sites where it has been described include the pleura [10], gastrointestinal tract, pancreas, and kidney [11]. We report a case of an INI1-deficient tumor of unknown origin proven with solid molecular biology data, confirming biallelic inactivation without any other genetic aberration on NGS.

The expression of cytokeratin on immunostains supports an epithelial origin rather than sarcomas or AT/RT. This emerging group of tumors in young adults, older than those seen in AT/RT, may lead to the definition of a new entity with the extensive use of immunohistochemistry testing.

We hereby report the first case of a neck lymph node metastasis with INI1-deficient carcinoma of unknown primary site with these particularities. Histologic and molecular analysis enabled us to complete the diagnosis assessment after initiating therapy. The initial therapy was performed with standard head and neck treatment. Another particularity of this case was the absence of tumor and lymph node metabolism on the FDG PET–CT examination, which is in contrast to the imaging appearance of SMARCB1 (INI1)-deficient sinonasal carcinoma described as having avidity to FDG on FDG PET–CT [12], as well as the rapid growth of this carcinoma. The complete staging report confirmed the diagnosis of neck node carcinoma with unknown primary. Considering the aggressive nature of this tumor, the therapeutic management included primary local therapy with surgery of the node disease through bilateral lateral and central node dissection, followed by chemotherapy due to the short interval of recurrence before radiation therapy. After six cycles of carboplatin and paclitaxel, there was no evidence of involvement.

In the literature, head and neck INI1-deficient locations in the adult population were represented by sinonasal carcinomas. Recently, a pooled analysis of sinonasal carcinomas recommended a multimodal therapeutic strategy to improve survival outcomes [13]. Different options for therapy are described, analogous to the sinonasal location. These options include surgery, when possible, combined with chemotherapy and radiation [2]; neoadjuvant chemotherapy, with the aim of decreasing tumor size before local therapy; and radical surgery followed by postoperative radiotherapy [7], leading to clinical remission. We recommended the first option because of rapid growth of the tumor with initial surgery followed by chemotherapy and radiation therapy. Locoregional and distant control were obtained 18 months after therapy.

In cases of INI1-deficient carcinoma, such as in the sinonasal region, tumors may be poorly differentiated or undifferentiated, making classification difficult. In the case of INI1-deficient carcinoma without a primary site, consistent histopathological findings were observed, including varying proportions of plasmacytoid/rhabdoid cells [2]. The absence of a primary location in this case complicated the diagnosis, histological finding, and classification due to the absence of epithelial and glandular elements. In the literature sinonasal INI1-deficient carcinoma had immunohistochemically diffused expression of cytokeratins (CK), p16, p40, and p63 in all cases, while expression of CK5/6, CK7 and vimentin was only focal or absent, [8] and the diagnosis of melanoma was definitively excluded. The patient had negative expression of p16, p40, and p53.

RNA-sequence analysis found a transcriptional fusion of SMARCB1/RP6-65G23.3 with biallelic inactivation and homozygote deletion of SMARCB1 gene. Loss of expression of SMARCB1 is described in the epithelioid sarcomas [14], epithelioid malignant peripheral nerve sheath tumors, and kidney medullary carcinomas [15]. We secondarily decided to perform a MRI of the kidney to formally eliminate a primitive kidney location because of the delay of assessment of RNA-sequence analysis. SMARCB1 deletion in this unusual situation of undifferentiated carcinoma should be completed because of the opportunity to offer new treatments. This molecular deletion will open the access of molecular therapy in case of distant metastasis using autophagy inhibitors or proteasome inhibitors combined with a cytotoxic chemotherapy currently evaluated in kidney medullary carcinomas [16].

## Conclusion

The complete extension report confirmed the complex diagnosis of an unusual undifferentiated carcinoma of the lymph nodes without a primary site. The unique feature of this tumor was the SMARCB1 deletion. Therefore, in cases of unusual presentation of neck node carcinoma in young patients without a primary site, molecular analysis should consider SMARCB1 deletion. The therapeutic sequence including surgery, chemotherapy, and radiotherapy should be discussed due to the rapid growth and stage of the tumor. Research of SMARCB1 deletion should be performed for undifferentiated carcinomas of young patients because of the possibility of molecular therapy, such as autophagy inhibitors or proteasome inhibitors, in clinical trials.

## Data Availability

All data can be found at the department of head and neck surgery of Institut Curie from Paris.
